# Magnitude and associated factors of preoperative anemia among adult elective surgical patients at Saint Paul’s Hospital Millennium Medical College, Addis Ababa, Ethiopia, 2024

**DOI:** 10.3389/fmed.2024.1466554

**Published:** 2024-12-16

**Authors:** Wondimnew Mersha Biset, Nura Nasser, Lemi Belay Tolu, Nuhamin Tesfa Tsega, Mebratu Abraha

**Affiliations:** ^1^Department of Anesthesiology, Critical Care and Pain Medicine, Saint Paul’s Hospital Millennium Medical College, Addis Ababa, Ethiopia; ^2^Department of Obstetrics and Gynecology, Saint Paul’s Hospital Millennium Medical College, Addis Ababa, Ethiopia; ^3^Department of Women’s and Family Health, School of Midwifery, College of Medicine and Health Sciences, University of Gondar, Gondar, Ethiopia; ^4^Reaserch Directorate Office and Nursing Education Department, Saint Paul’s Hospital Millennium Medical College, Addis Ababa, Ethiopia

**Keywords:** magnitude, associated factors, preoperative anemia, adult elective surgical patients, Ethiopia

## Abstract

**Background:**

Preoperative anemia is a common hematologic public health problem among elective surgical patients. Preoperative anemia complications independently increase the risk of perioperative complications and mortality rate. Despite this complication, there is a scarcity of evidence on the prevalence and associated factors of preoperative anemia among adult elective surgical patients in Ethiopia. Therefore, this study aimed to investigate the magnitude and associated factors of preoperative anemia among adult elective surgical patients at Saint Paul’s Hospital Millennium Medical College, Addis Ababa, Ethiopia.

**Methods:**

An institution-based cross-sectional study was conducted from January 1 to 30, 2024 at Saint Paul’s Hospital Millennium Medical College. A consecutive sampling technique was used to select eligible study participants. Chart review and a structured, pretested, and interviewer-administered questionnaire were employed. Kobo toolbox was used to collect the data and exported to SPSS version 25 software for data cleaning, coding, and analysis. Binary logistic regression model was fitted to identify factors associated with preoperative anemia. An adjusted odds ratio (AOR) with its 95% CI and a *p*-value of ≤0.05 was used to declare a statistical association.

**Results:**

A total of 247 study participants were included in the analysis, giving a response rate of 98.8%. Majority (68%) of the participants were from urban areas. The median age of the patient was 38 (IQR ± 32) years. The magnitude of preoperative anemia was found to be 27.1% (95% CI: 21.9, 33.1). Being female (AOR = 3.44; 95%CI: 1.53, 7.73), being overweight (AOR = 0.26; 95%CI: 0.10, 0.88), and having infection and injury/trauma as indications for surgery (AOR = 4.59; 95%CI: 1.62, 12.96) and (AOR = 3.58; 95%CI: 1.35, 9.49) were significantly associated with preoperative anemia.

**Conclusion:**

In this study, more than one-fourth of the study participants had preoperative anemia. To decrease this public health problem, it is better to screen at risk patients, specifically females and trauma patients during the preoperative visit and disseminate information about a healthy diet and the prevention and treatment of anemia through health education.

## Background

Anemia is a global public health problem affecting both developing and developed countries, with major consequences for human health as well as social and economic development (1). As defined by the World Health Organization (WHO), anemia is a condition in which the number of red blood cells or the hemoglobin concentration within them is lower than the normal range, which decreases the oxygen-carrying capacity of red blood cells to tissues. According to WHO classification criteria, anemia is classified as mild, moderate, and severe based on the concentrations of hemoglobin in the blood, and its classification varies according to age and gender (2). In 2019, around 1.8 billion individuals globally were affected by anemia (3). According to the 2016 Ethiopia demographic and health survey, the prevalence of anemia in Ethiopia was 23.6 and 14.5% in adult women and men, respectively (4). Anemia has pathophysiological diverse effects and has multifactorial causes (3, 5).

Preoperative anemia is an emerging concern in patients scheduled for surgical procedures (6). It is more frequent in surgical patients as compared with the general population, and its magnitude can reach 30–40%, even in some setups to 75% depending on different factors (7). The global estimate of preoperative anemia among surgical patients was around 35% (8).

Empirical evidence shows that preoperative anemia is associated with increased postoperative morbidity and mortality (9–12). It also increases the risk of post-operative complications, including unexpected Intensive Care Unit (ICU) admission (11, 13), hospital length of stay (6, 11, 14), surgical site infection (15), and hospital readmission (13, 16, 17). Moreover, it elevates the risk of blood transfusion (18), cardiac infarction, renal failure, stroke, death (7, 19), and increases health care resource use (11, 16).

Different evidence show that there are different causes that are attributable to preoperative anemia. Among these are hospital-acquired anemia, iron deficiency anemia, anemia of chronic renal disease, underling malignancy, and chronic illness (20–22). Preoperative anemia is a preventable risk for surgical patients, and its treatment reduces the need for blood transfusions while also improving patient outcomes (23). Preoperative anemia management results in higher pre-operative hemoglobin concentrations and less need for blood transfusions (22).

Findings from published studies revealed that different factors such as the age of the patient (24, 25), sex (24–26), residence (27), American Society of Anesthesiology Physical Status (ASA-PS) classification (24, 28, 29), history of recent prior surgery (28), indication for surgery (29, 30), type of surgery (25, 28), history of malignancy (28, 31), HIV (30, 32), chronic kidney disease (31), asthma (29, 33), chemotherapy/radiotherapy use (34, 35), use of Highly Active Antiretroviral Therapy (HAART) (36–38), and Non-steroidal Anti-Inflammatory Drugs (NSAIDS) utilization (39, 40) were found to be significantly associated with preoperative anemia.

Optimizing preoperative hemoglobin, including the treatment of iron deficiency anemia, is recommended before elective surgery (6, 22). Even though it is a treatable disease, it remains a neglected public health issue in Ethiopia. As a result, determining the magnitude and associated factors of preoperative anemia will be critical for improving postoperative patient outcomes. In Ethiopia, various studies on anemia have been conducted in the general population, but there is a scarcity of evidence on the magnitude and associated factors of preoperative anemia among adult elective surgical patients. Therefore, the aim of this study was to assess the magnitude and associated factors of preoperative anemia among adult elective surgical patients at Saint Paul’s Hospital Millennium Medical College (SPHMMC) by filling such research gaps and incorporating important variables that previous studies missed.

## Methods

### Study design and period

An institution-based cross-sectional study was conducted from January 1 to 30, 2024.

### Study setting

The study was employed at SPHMMC, which is found in Gulele sub-city, Addis Ababa, the capital city of Ethiopia, and one of the biggest tertiary hospitals in Addis Ababa. In addition to being a tertiary referral hospital, it provides medical, surgical, pediatric, and obstetrical services on an elective and emergency basis. It is the only hospital in Ethiopia to provide renal transplantation and infertility services. Based on the hospital’s data, on average, around 618 patients are scheduled for elective surgery each month. Saint Paul’s Hospital Millennium Medical College has 18 major elective functional operating rooms, including orthopedics, urology surgery, gynecology, pediatrics, general surgery, plastic, otolaryngology, maxillofacial, ophthalmology, cardiothoracic, and neurosurgery theater rooms.

### Source and study population

In this study, the source population included all adult patients who are scheduled for elective surgery at SPHMMC, while all adult patients who are scheduled for elective surgery at SPHMMC during the study period were our study population.

### Eligibility criteria

The inclusion criteria was that all adult patients who were scheduled for elective surgery at SPHMMC were included. Known anemic patients who were on treatment, patients who came for day-case surgery (because it is unethical to send for hemoglobin measurement solely for research purpose), and obstetric patients (due to physiological changes during pregnancy can affect both the risk factors and cutoffs of hemoglobin to diagnose anemia) were excluded from this study. Besides, patients who rescheduled for repeated surgery during the data collection period were excluded.

### Sample size determination and sampling procedure

The sample size was determined using the single population proportion formula by considering the following assumptions: the proportion of preoperative anemia in the previous study was 36.8% (28), 95% confidence level, and 5% margin of error.

(n) $=\frac{{\left(Z\alpha /2\right)}^{2}p\left(1-p\right)}{d^{2}}$ $\frac{={\left(1.96\right)}^{2*}0.368\left(1-0.368\right)}{\left(0.05\right)2}$ = 357

Where; n = the desirable sample size, Z a/_2_ = standard normal distribution curve value for 95% confidence level = 1.96, P = proportion of preoperative anemia, and d = 5% margin of error.

Since the source population was less than 10,000, the sample size correction formula was applied as follow; Nf= $\frac{ni}{1+\frac{ni}{N}}$ = Nf= $\frac{357}{1+\frac{357}{618}}$ Nf = 227

Where, ni = calculated sample size

N = source population = 618

**Figure 1:**
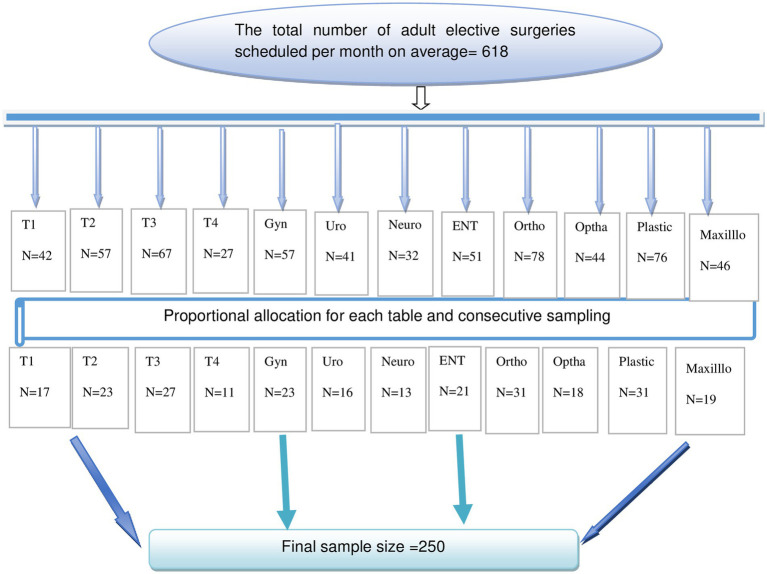
Schematic presentation of sampling procedure.

Therefore, by considering a 10% non-response rate, the minimum required sample size was 250. The total calculated sample size was proportionally allocated for each table (T1–T4, gynecology, urology, ophthalmology, plastic, maxillofacial, ENT, neurology, and orthopedics) depending on their load of schedule (Figure 1). The study participants in each table were selected using a consecutive sampling technique. All the study participants were included in this study until the required sample size was achieved during the study period.

### Study variables

Preoperative anemia was the outcome variable, whereas sex, age, residence, marital status, educational status, occupational status, media exposure, Body Mass Index (BMI), ASA-PS status, history of malignancy, HIV, diabetes mellitus, history of chronic kidney disease, smoking, history of malaria, asthma, history of recent prior surgery, indication for surgery, use of chemotherapy, use of NSAIDS, and use of HAART were independent variables.

### Operational definition

Anemia: according to WHO definition, it is defined as a reduction of hemoglobin level below 12 g/dL (hematocrit <36%) for non-pregnant women and 13 g/dL (hematocrit <39%) for men (2).

Mild anemia is classified as hemoglobin between 11–11.9 g/dL and 11–12.9 g/dL for female and male, respectively (2).

Severe and moderate anemia: hemoglobin measurements less than 8 g/dL and between 8–10.9 g/dL are classified as severe and moderate anemia, respectively for both male and female (2).

Preoperative anemia: defined as a reduction of hemoglobin level below 12 g/dL (hematocrit <36%) for non-pregnant women and 13 g/dL (hematocrit <39%) for men in the preoperative period.

Adult patient: defined as age greater than 18 years old (12, 21, 31).

Media exposure: was defined as if patients had been exposed to at least one of the three media (television, radio, or newspaper) were considered to be exposed and otherwise unexposed (41).

Recent prior surgery is defined as any major surgical operation done within 2 months before the current surgery (28).

### Data collection tools and procedures

The data collection tool was developed by reviewing different literatures (24, 26, 28–31, 34–36). Data was collected using both chart reviews and a structured and interviewer-administered questionnaire through face-to-face interviews. The questionnaire contains socio-demographic, clinical, hematologic, and medication-related variables. Two BSc Nurse data collectors and one Anesthesiology resident supervisor received training regarding the purpose of the study, data collection methods, participant approaches, interviewing strategies, and information retention.

### Data quality control

Initially, to assure the quality of the data, two days of training was given to the data collectors and supervisor. The questionnaire was prepared in English version and translated to Amharic language and back to English to ensure its consistency and better understandability. Besides, before the actual data collection, a pretest was done on 5% of the sample size to check the response, language clarity, and appropriateness of the questionnaire. Thereafter, amendments were made accordingly. During the actual data collection, the supervisor and principal investigator closely followed the day-to-day data collection process and ensured the completeness and consistency of the collected data.

### Data processing and analysis

Data was collected using Kobo Toolbox and exported to SPSS version 25 for data cleaning, coding, and analysis. Descriptive statistics like percentages, frequency tables, and graphs were used to present the characteristics of the study participants. Binary logistic regression was fitted to identify associated factors for preoperative anemia. Initially, bivariable analysis was done to identify the eligible independent variables and variables with a *p*-value of ≤ 0.25 were included in the multivariable logistic regression analysis. In the multivariable logistic regression analysis, a *p*-value of ≤ 0.05 with a 95% CI for the adjusted odds ratio (AOR) was used to declare the level of significance. Model fitness for the final model was checked using Hosmer and Lemeshow goodness of fit and found to be fitted. Besides, Multicollinearity was checked using the variance inflation factor (VIF), which indicates that there was no Multicollinearity.

### Ethical consideration

Ethical approval was obtained from the research directorate of SPHMMC Institutional Review Board (IRB) (reference number: PM23/365). Written informed consent was obtained from each study participant after a clear explanation of the objective of the study. Patients having hemoglobin level less than 7 were referred for blood transfusion, but data was collected before transfusion. To assure confidentiality and privacy, data were coded, and names were not included in the data collection format. After entering the computer, the data was not disclosed to anyone other than the principal investigator.

## Results

### Sociodemographic characteristics of the study participants

A total of 247 patients were included in this study, making a response rate of 98.8%. Among these, 51% were male and 68% of the respondents were from urban areas. The median age of the patients was 38 (IQR ± 32) years old, and 35.2% were aged 30–49 years. Around 25.5% of the study participants had no formal education. Besides, 81.4% of the respondents had media exposure (Table 1).

**Table 1:** Sociodemographic characteristics of the study participants among adult elective surgical patients at Saint Paul’s Hospital Millennium Medical College, Addis Ababa, Ethiopia, 2024.

Variables	Categories	Frequency	Percentage
Sex	Male	126	51
	Female	121	49
Residency	Urban	168	68
	Rural	32	32
Age	18–29	74	30
	30–49	87	35.2
	50–69	61	24.7
	70 and above	25	10.1
Current marital status	Married	138	55.9
	Unmarried	109	44.1
Educational status	Have no formal education	63	25.5
	Primary education	47	19
	Secondary education	81	32.8
	College and above	56	22.7
Occupational status	Government employee	54	21.9
	Self-employee	79	32.0
	Others	68	27.5
	No job	46	18.6
Media exposure	Unexposed	46	18.6
	Exposed	201	81.4

### Clinical and medication related characteristics of the study participants

Among the study participants, 47.4 and 66.4% of the participants had ASA-PS II and normal BMI, respectively. Regarding recent prior surgery, 81.4% of the respondents had no recent prior surgery. Approximately 1/10th (8.1%) of the participants had DM. Ninety-six percent of the participants have no history of cigarette smoking. About 10.1% of the patients were using NSAIDS. Moreover, 97.2% of the study participants had no history of chemotherapy use (Table 2).

**Table 2:** Clinical and medication related characteristics of the study participants among adult elective surgical patients at Saint Paul’s Hospital Millennium Medical College, Addis Ababa, Ethiopia, 2024.

Variables	Category	Frequency	Percentage
ASA-PS classification	I	114	46.1
	II	117	47.4
	III and above	16	6.5
BMI	<18.5	31	12.6
	18.5–24.9	164	66.4
	25 and above	52	21.1
Recent prior surgery	Yes	46	18.6
	No	201	81.4
Malarial attack	Yes	22	8.9
	No	255	91.1
Cigarette smoking	Yes	10	4
	No	237	96
HIV status	Negative	195	78.9
	Positive	7	2.8
	Not screened	45	18.2
DM	Yes	20	8.1
	No	227	91.9
Asthma	Yes	7	2.8
	No	240	97.2
Peptic ulcer disease	Yes	21	8.5
	No	91.5	91.5
Malignancy	Yes	18	7.3
	No	229	92.7
Indication of surgery	Non infectious	173	70
	Infectious	34	13.8
	Injury/trauma	40	16.2
Type of surgery	General	45	18.2
	Gynecology	22	8.9
	Cardiothoracic	11	4.5
	Hepatobiliary	22	8.9
	Urologic	16	6.5
	Orthopedic	30	12.1
	Plastic	30	12.1
	Neurologic	13	5.3
	ENT	21	8.5
	Maxillofacial	19	7.7
	Ophthalmology	18	7.3
Admitted for the last 1 month	Yes	50	20.2
	No	197	79.8
Diagnosed chronic kidney disease	Yes	6	2.4
	No	241	97.6
History of chemotherapy use	Yes	7	2.8
	No	240	97.2
NSAIDS	Yes	25	10.1
	No	222	89.9
HAART	Yes	7	2.8
	No	240	97.2

### Magnitude of preoperative anemia among elective surgical patients

**Figure 2:**
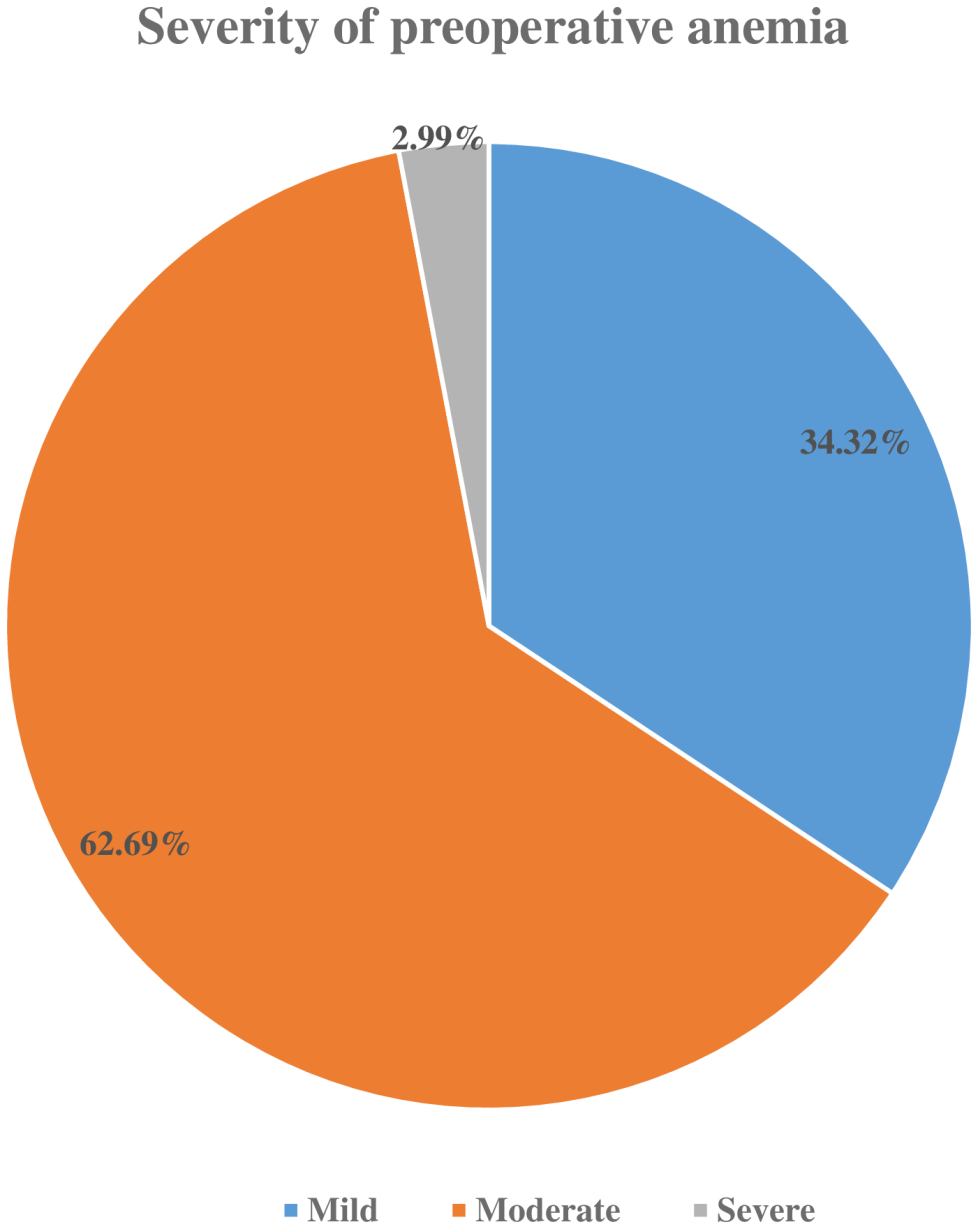
Severity of preoperative anemia among adult elective surgical patients at Saint Paul’s Hospital Millennium Medical College, Addis Ababa, Ethiopia, 2024.

In this study, the overall magnitude of preoperative anemia was found to be 27.1% (95%CI: 21.9, 33.1). Among anemic patients, 62.69% had moderate preoperative anemia (Figure 2).

### Factors associated with preoperative anemia

In the bivariable logistic regression analysis, sex, residency, age, educational status, occupation status, media exposure, BMI, recent prior surgery, malaria attack, indication of surgery, and admission for the last 1 month were found to have a *p*-value of ≤0.25 and were considered for the multivariable logistic analysis. However, sex, BMI, and indication of surgery were significantly associated with preoperative anemia among adult elective surgical patients in the multivariable analysis.

In this study, being female had 3.44 (AOR = 3.44; 95CI: 1.53, 7.73) times higher odds of preoperative anemia than male respondents. The odds of preoperative anemia among patients whose BMI ≥25 were 74% (AOR = 0.26; 95%CI: 0.10, 0.88) less likely than patients whose BMI was 18.5–24.9. Regarding indication for surgery, patients whose indications for surgery were infection and injury/trauma were 4.59 (AOR = 4.59; 95%CI: 1.62, 12.96) and 3.58 (AOR = 3.58; 95%CI: 1.35, 9.49) times more likely to experience preoperative anemia, respectively, as compared with their counterparts (Table 3).

**Table 3:** Bivariable and multivariable logistic regression analysis of associated factors of preoperative anemia among adult elective surgical patients at Saint Paul’s Hospital Millennium Medical College, Addis Ababa, Ethiopia, 2024.

Variables	Preoperative anemia		COR (95%CI)	AOR (95%CI)
	Yes	No		
Sex				
Male	27	99	1	1
Female	40	81	1.81 (1.02,3.20)	3.44 (1.53, 7.73)**
Residency				
Urban	35	133	1	1
Rural	32	47	2.59 (1.44, 4.64)	1.05 (0.40, 2.75)
Age				
18–29	26	48	1	1
30–49	21	66	0.59 (0.29, 1.17)	1.01 (0.42, 2.43)
50–69	12	49	0.45 (0.21, 0.99)	0.89 (0.29, 2.74)
70 and above	8	17	0.87 (0.33, 2.28)	1.54 (0.36, 6.60)
Educational status				
Have no formal education	22	41	2.80 (1.16, 6.77)	2.11 (0.52, 8.51)
Primary education	9	38	1.24 (0.45, 3.42)	1.01 (0.28, 3.67)
Secondary education	27	54	2.61 (1.12, 6.12)	1.88 (0.62, 5.75)
College and above	9	47	1	1
Occupation				
Government employee	10	44	1	1
Self-employee	25	54	2.04 (0.89, 4.69)	1.25 (0.42, 3.72)
Others	24	44	2.40 (1.03, 5.60)	0.98 (0.29, 3.30)
No job	8	38	0.93 (0.33, 2.58)	0.39 (0.10, 1.54)
Media exposure				
Exposed	49	152	1	1
Un exposed	18	28	1.99 (1.02, 3.91)	2.59 (0.86, 7.78)
BMI				
<18.5	10	21	1.03 (0.45, 2.33)	1.02 (0.37, 2.81)
18.5–24.9	52	112	1	1
25 and above	5	47	0.23 (0.09, 0.61)	0.26 (0.10, 0.88)*
Recent prior surgery				
Yes	28	18	6.46 (3.25, 12.85)	2.63 (0.94, 7.30)
No	39	162	1	1
Malaria attack				
Yes	11	11	3.02 (1.24, 7.34)	2.98 (0.96, 9.23)
No	56	169	1	1
Indication for surgery				
Non infectious	30	143	1	1
Infectious	20	14	6.81 (3.10, 14,98)	4.59 (1.62, 12.96)**
Injury/trauma	17	23	3.52 (1.68, 7.39)	3.58 (1.35, 9.49)*
Admitted for the last 1 month				
Yes	28	39	5.16 (2.67, 9.97)	1.75 (0.64, 4.77)
No	22	158	1	1

* = *P*-value < 0.05, ** = *P*-value < 0.01 BMI; Body Mass Index.

## Discussion

This institution-based cross-sectional study assessed the magnitude of preoperative anemia and its associated factors among adult elective surgical patients at SPHMMC, Addis Ababa. Thus, it was found that the magnitude of preoperative anemia was 27.1% (95% CI: 21.9, 33.1). This is consistent with studies conducted in Singapore- 27.8% (24) and Germany- 32.5% (42).

However, the finding of this study was higher as compared to studies conducted in USA-12.5% (43) and Australia-13.9% (21). The possible explanation for this variation might be due to the difference in the study setting and the study population. In this study, we used all types of elective surgical patients, including orthopedic patients, which might increase the prevalence of preoperative anemia because of active blood loss (28). In contrast, the study population in the USA was only thyroid cancer patients. Besides, the study population in the USA and Australia might have different awareness and pre-screening behaviors about anemia and having good dietary intake, which decreases the risk of anemia (44).

On the other hand, the prevalence of this study was lower than studies done at Gondar University Hospital-36.8% (28), South Africa-47.8% (29), and Spain-36% (45). The possible explanations could be the differences in the characteristics of the study participants and the outcome variable cut-off point. In this study, nearly one-third (32%) of the study respondents were from rural areas, whereas a study done at University of Gondar, 58.9% of the study participants were from rural areas. This disparity indicates that rural residents had a higher risk of developing anemia in the preoperative period (46). The study done in Spain used the same cutoff point for both male and female with 13 g/dL to declare preoperative anemia. However, in this study, we used different cutoff points with hemoglobin level below 12 g/dL (hematocrit <36%) for non-pregnant women and 13 g/dL (hematocrit <39%) for men.

Regarding factors associated with preoperative anemia, this study found that there was a significant association between sex of patients and preoperative anemia. The odds of having preoperative anemia among female patients were 3.44 times higher as compared with their male counterparts. Similar findings were reported from previous studies done in Ghana (26) and China (25). The possible justification might be that women are biologically vulnerable to the inevitable iron loss during menstruation, childbirth, and lactation in the reproductive age, which may cause anemia in the preoperative period (47, 48).

Consistent with studies conducted in Mali (49), Bangladesh (50), China (51), and Colombia (52), BMI was significantly associated with preoperative anemia. Patients who had ≥25 BMI had 74% (AOR = 0.26; 95%CI: 0.10, 0.88) lower odds of preoperative anemia than patients who had 18.5–24.9 BMI. The possible explanations might be that patients with a higher BMI may have had good dietary intake and higher iron consumption as compared to their counterparts.

In this study, the indication for surgery was significantly associated with preoperative anemia. Patients whose indications for surgery were infection and injury/trauma were 4.59 (AOR = 4.59; 95%CI: 1.62, 12.96) and 3.58 (AOR = 3.58; 95%CI: 1.35, 9.49) times more likely to experience preoperative anemia, respectively, as compared with patients whose indication for surgery was non-infectious. This result is supported by study conducted in South Africa (30). This could be due to the fact that infection can depress bone marrow, erythropoietin synthesis, decrease intestinal iron absorption, and decrease release from body iron stores and transport (53). Besides, injury/trauma leads to anemia in the preoperative period due to acute blood lose and reduce erythropoiesis due to injury/trauma-associated inflammation (54).

The authors strongly believe that this study is very important in providing evidence about the magnitude and associated factors of preoperative anemia among adult elective surgical patients. Based on this evidence, policymakers should consider the burden of preoperative anemia, and health care providers should focus on preoperative optimization of preoperative anemia. Lastly, we authors would like to acknowledge the limitation of this study. Due to the cross-sectional nature of the study, we are unable to establish a causal relationship between preoperative anemia and the identified independent variables. Besides, we did not assess the effect of preoperative anemia on patient outcomes.

## Conclusion

In this study, more than 1/4th of the study participants had preoperative anemia. Being female and having infection and injury/trauma as indications for surgery increased the odds of having preoperative anemia among adult elective surgical patients, while being overweight (BMI ≥25) decreased the odds of having preoperative anemia among adult elective surgical patients.

To decrease this public health problem, it is better to routinely practice postponement of purely elective surgeries for optimization. Special attention shall be given to at-risk patients (female, patients whose indications for surgery were infection, trauma/injury). In addition, it is better to disseminate information about a healthy diet and the prevention and treatment of anemia through health education. For future researchers, we recommend larger samples to confirm findings, especially for the specific subpopulations, and a prospective cohort study to assess the effect of preoperative anemia on patient outcomes.

## Data Availability

The original contributions presented in the study are included in the article/Supplementary material, further inquiries can be directed to the corresponding author.
