# Supplementary material for: Magnitude and associated factors of preoperative anemia among adult elective surgical patients at Saint Paul’s Hospital Millennium Medical College, Addis Ababa, Ethiopia, 2024
Source: Front Med (Lausanne). 2024 Dec 16;11:1466554. doi: 10.3389/fmed.2024.1466554 (PMC11683085; doi:10.3389/fmed.2024.1466554)
Supplement: Supplementary file 1 [file Table_1.DOCX]

**English version questionnaires**

| S.No | Questionnaire | Possible answers | Skip code |
| --- | --- | --- | --- |
| **Part I. Sociodemographic characteristics** | | | |
| 101 | How old are you? | ________ in year |  |
| 102 | Sex of respondent | 1. Male 2. Female |  |
| 103 | Where do you live? | 1. Urban 2. Rural |  |
| 104 | What is your current marital status? | 1. Single 2. Married 3. Divorced 4. Widowed |  |
| 105 | What is your educational status? | 1. Unable to read and write 2. Able to read and write 3. Primary education 4. Secondary education 5. College and above |  |
| 106 | What is your occupational status? | 1. House wife 2. Merchant 3. Government employee 4. Self employed 5. Student 6. No job 7. Others (specify)…………… |  |
| 107 | Have you ever read newspapers? | 1. Yes 2. No | If No, skip to Q 109 |
| 108 | If yes for Q,#108, how often did you read? | 1. Every day 2. One times per week 3. Two times per week 4. Three and more |  |
| 109 | Have you ever listened radio? | 1. Yes 2. No | If No, skip to Q 111 |
| 110 | If yes for Q,#110, how often did you listen? | 1. Every day 2. One times per week 3. Two times per week 4. Three and more per week |  |
| 111 | Have you ever watched a television? | 1. Yes 2. No | If No, skip Q 112 |
| 112 | If yes for Q,#112, how often did you watch? | 1. Every day 2. One times per week 3. two times per week 4. Three and more per week |  |
| **Part II. Clinical characteristics** | | | |
| 201 | Weight | _____in kg |  |
| 202 | Height | _____in cm |  |
|  | BMI | ________Kg/m^2^ |  |
| 203 | ASA-PS classification of the respondent | 1. I 2. II 3. III 4. IV 5. V |  |
| 204 | Do you have any surgery in the past 2 months? | 1. Yes 2. No |  |
| 205 | Have you had history of malarial attack? | 1. Yes 2. No |  |
| 206 | Do you smoke cigarette | 1. Yes 2. No |  |
| 207 | HIV status | 1. Positive 2. Negative 3. Not screened |  |
| 208 | Diabetes mellitus | 1. Yes 2. No |  |
| 209 | Asthma | 1. Yes 2. No |  |
| 210 | History of peptic ulcer disease | 1. Yes 2. No |  |
| 211 | Having Malignancy | 1. Yes 2. No |  |
| 212 | Indication of surgery | 1. Non infectious  2. Infectious  3. injury/trauma |  |
| 213 | Type of surgery | 1. General 2. Gynecologic 3. Cardiothoracic 4. Hepatobiliary 5. Urologic 6. Other |  |
| 214 | Have you been admitted for the last 1 month? | 1. Yes 2. No |  |
| 215 | History of chronic kidney disease | 1. Yes 2. No |  |
| **Part III. Medication related factors** | | | |
| 301 | History of use of chemotherapy | - - - 1. Yes  1. No |  |
| 302 | Use of HAART | 1. Yes 2. No |  |
| 303 | Use of NSAIDS | 1. Yes 2. No |  |
| **Part IV. Measurement of outcome variable** | | | |
| 401 | Hemoglobin level | ____mg/dl |  |
